# Neurodegenerative disorders: Stereotyped relationships between pathological metabolic topographies and the healthy default mode network

**DOI:** 10.21203/rs.3.rs-11320531/v1

**Published:** 2026-10-13

**Authors:** Matej Perovnik, David Eidelberg

## Abstract

**Background and Objectives:** Neurodegenerative disorders are associated with disease-specific networks on metabolic brain imaging. Depending on the disease, the associated pathological topography overlaps to varying degrees with the healthy default mode network (DMN). In this study, we examined the relationships of these networks in various neurodegenerative disorders and in prodromal syndromes. **Methods:** We analyzed FDG PET scans from 1,117 patients with neurodegenerative disorders manifest by dementia (Alzheimer’s disease (AD), behavioral variant frontotemporal dementia (bvFTD), and dementia with Lewy bodies (DLB)), parkinsonism (Parkinson’s disease (PD) and PD dementia (PDD)), or by mixed/atypical syndromes (progressive supranuclear palsy (PSP), multiple system atrophy (MSA), corticobasal degeneration (CBD), and X-linked dystonia-parkinsonism (XDP)). We also studied scan data from 120 individuals with prodromal syndromes. Topographic relationships between the disease-specific networks and the healthy DMN were assessed by computing spatial similarity indices and voxel-wise correlations. We also computed the expression of the two networks in each patient and in 499 healthy subjects. In each patient group, the resulting values were correlated with each other and with corresponding MMSE scores. **Results:** The disorders fell into one of three categories based on the topographic relationship of the disease-specific and healthy networks. Category 1 (bvFTD, PSP, CBD, and XDP) was characterized by substantial spatial overlap of the two metabolic networks, tight correlations between their respective expression levels, and significant DMN reductions. Category 2 (PD, AD, DLB, and their associated prodromal syndromes) was characterized by less pronounced overlap, correspondingly weak correlations, and smaller DMN reductions, particularly at prodromal stages. In this group, correlations with MMSE were greater for the difference between disease network and DMN scores than for the disease network alone. Category 3 (MSA) had no discernible network overlap and no loss of DMN expression. **Discussion:** Category 1 disorders were associated with close relationships between the pathological and healthy topographies, indicating primary DMN involvement. Category 2 disorders were characterized by weaker network relationships, consistent with secondary DMN involvement. Category 3 disorders showed no evidence of DMN involvement. The degree to which disease-specific networks overlap with DMN provides unique insight into the underlying neurodegenerative process.

### Introduction

Neurodegenerative diseases are characterized by the pathological accumulation of misfolded proteins, which play pivotal roles in the development of clinical manifestations. For instance, α-synuclein accumulates in Parkinson's disease (PD), dementia with Lewy bodies (DLB), and multiple system atrophy (MSA). Amyloid β and a mixture of three and four microtubule-binding repeats (3R and 4R) tau are primary aggregates in Alzheimer's disease (AD). While corticobasal syndrome can be a clinical manifestation of AD, it is more frequently associated with the characteristic pathological changes of corticobasal degeneration (CBD) including 4R tau accumulation. The latter is also the main culprit in progressive supranuclear palsy (PSP), and either 4R tau or TAR DNA binding protein 43 (TDP-43) in behavioral variant frontotemporal dementia (bvFTD).[1] Interestingly, other examples of tau-related neurodegenerative disorders have been noted, such as X-linked dystonia-parkinsonism (XDP), a rare X-linked recessive disorder endemic to Panay Island in the Philippines. This disorder has been linked to deposition of hyperphosphorylated 4R tau isoforms in the striatum and other brain regions.[2] Whether brain networks serve as conduits for the spread of misfolded proteins along stereotyped pathways and/or have a critical role in determining clinical phenotype is not known.[3] It is nonetheless clear that neurodegenerative diseases are associated with marked changes in normal resting-state networks (RSNs) as well as the appearance of abnormal functional brain networks that emerge as the disease progresses.[4,5]

#### *The metabolic default mode network*

The default mode network (DMN) refers to a set of highly interconnected regions (nodes) positioned at the end of the processing streams in the parietal, temporal, and frontal cortex.[6,7]

Since its identification in the 1990s, the DMN has been the focus of numerous studies in health as well as brain disease.[8] Under normal circumstances, the DMN is involved in many aspects of cognition, including the integration of memory, language, and semantic memory processes as well as the sense of self.[8,9] By contrast, in neurological and psychiatric disorders, DMN dysfunction has been linked to a variety of cognitive syndromes.[10] Indeed, the dominant position of the DMN relative to the other large-scale brain networks makes it a central computational hub for cognitive integration in the human brain.[6,9]

*Disease-specific metabolic topographies*

Neurodegenerative disorders are characterized by abnormal networks unraveled through linear statistical methods such as principal component analysis (PCA) and independent component analysis (ICA) applied to functional imaging data, acquired with $^{18}$F-fluorodeoxyglucose (FDG) PET or resting-state functional MRI (rs-fMRI).[5] Since the earliest description of specific spatial covariance patterns associated with neurodegenerative disorders, substantial effort has been dedicated to the characterization of the resulting disease-related topographies and their relationship to underlying pathology.[11–13] In addition to the study of group-level changes, one can measure the expression of a known network in an individual by computing the scalar product of the subject's regional data vector and the generic disease pattern.[14] The resulting subject score thus represents the expression of a validated disease network in a given subject's scan. These values have been successfully utilized to improve diagnostic and prognostic accuracy[15,16] and to track disease progression and treatment responses in longitudinal imaging data.[17,18] Importantly, progressive increases in disease network expression have been shown consistently to reflect advancing disease in cross-sectional and longitudinal studies.[5,19,20] Disease-related networks are characterized by unique topographical representations that are pathologically specific (Figure 1A).[5,12] Even so, disease-specific networks when considered in isolation are unlikely to capture important interactions with healthy networks that may also be present.

In the current study, we aimed to gain further insight into the network pathology of neurodegenerative disorders by evaluating the state of the healthy DMN in relation to the emerging disease topography. We hypothesized that specific neurodegenerative disorders would show one of three characteristic network signatures: (i) Close spatial correspondence of the disease-specific topography with the healthy DMN and correspondingly strong inverse correlations between their respective subject scores. DMN expression will be reduced early in the disease course in these patients. (ii) Moderate spatial overlap of the disease-specific and healthy topographies, with weaker correlations between the corresponding subject scores. Reductions in healthy DMN expression will become prominent at later disease stages. (iii) No spatial overlap between the pathological and healthy topographies, and no correlation between the respective subject scores. Significant reduction in healthy DMN expression is not evident even at late-stage disease.

### Methods

#### *Participants*

We analyzed imaging data from 1,237 participants with various neurodegenerative disorders and 499 healthy control subjects (HCs). We included 239 individuals with PD, 213 with AD, 161 with DLB, 124 with MSA (including both MSA-P and MSA-C subtypes), 114 with PSP, 109 with FTD, 79 with mild cognitive impairment due to AD (MCI-AD), 69 with CBD, 58 with PD dementia (PDD), 30 with X-linked dystonia-parkinsonism (XDP), 28 with MCI with Lewy bodies (MCI-LB) and 13 with idiopathic REM sleep behavior disorder (iRBD) from 13 independent imaging centers and from a separate multicenter scan database. HCs were recruited from six independent centers and from two separate scan databases: the Alzheimer's Disease Neuroimaging Initiative (ADNI) and the Associazione Italiana Medicina Nucleare (AIMN). The details of the subjects in each group and imaging site are summarized in Supplementary Table 1. Baseline FDG PET data from the 79 MCI-AD patients were identified in the ADNI database as individuals with MCI and the AD CSF profile (p-$tau_{181}$/$A\beta_{42}$ > 0.021) who subsequently progressed to frank dementia at follow-up.

*Image preprocessing and network quantification*

Each subject's scan was realigned and spatially normalized to a standard Montreal Neurological Institute (MNI) PET template and smoothed with an isotropic Gaussian kernel (10 mm FWHM). To ensure compatibility with the original network maps, the image processing was performed using Statistical Parametric Mapping (SPM5) software (Wellcome Trust Centre for Neuroimaging, Institute of Neurology) running on Matlab R2021b (Mathworks Inc., Natick, MA). Scan data were logarithmically transformed and double-centered as a part of the forward application routine in ScAnVP (software available at: https://feinsteinneuroscience.org/) to calculate all subjects' DMN expression scores.[6] Additionally, for each group, expression levels (subject scores) for the relevant disease network (e.g., bvFTDRP for bvFTD; PDRP for PD, PDD, and iRBD; ADRP for AD and MCI-AD, etc.) were calculated on an individual case basis (see Supplementary Table 2). The resulting scores were standardized using local HC values. When specific local HC values were not available, as was the case in the large ADNI and AIMN cohorts, standardization was performed using the combined distribution of control values.

Outliers in each group/pattern were identified using Tukey's method. For the healthy DMN and the individual disease-specific topographies, the first (Q1) and third quartiles (Q3) were calculated and the interquartile range (IQR) was defined as the difference between Q3 and Q1. Observations falling below Q1−1.5×IQR or above Q3+1.5×IQR were considered to be outliers and were removed. In total, we excluded under 4% of scans in the patient and control groups (10 PD, 11 AD, 5 DLB, 4 MSA, 2 PSP, 9 bvFTD, 0 MCI-AD, 2 CBD, 2 PDD, 0 XDP, 0 MCI-LB, 0 iRBD, and 8 HC) from the final analyses.

*Topographic analyses*

The spatial overlap of the individual disease-specific networks with the healthy DMN topography was assessed using the Dice-Sørensen coefficient (DSC) of similarity.[21] To compute DSC, the DMN map was inverted (multiplied by -1) so that the corresponding positive and negative deviations reflected areas of homology on the two topographies. Both maps were thresholded at $|z| \geq 1$ within an explicit whole-brain mask to exclude out-of-brain voxels. The DSC was calculated from suprathreshold voxels, with overlap defined as voxels showing concordant signs (positive-positive or negative-negative) in both maps. Given the continuous distribution of the voxel weights on the disease and healthy networks, we also computed voxel-wise Pearson product-moment correlation coefficients (VWC) for each pair of topographies, corrected for spatial autocorrelation.[22]

*Statistics*

To test for differences in DMN and disease-related pattern expression between patients and age- and sex-matched groups of HC, we used Welch's two-sample *t*-tests. *P*-values were adjusted for multiple comparisons using the Bonferroni method; adjusted $p < 0.05$ were considered statistically significant. For each disease group, matched HCs were selected by 1:1 nearest-neighbor propensity-score matching on age and sex without replacement. This was done using the *MatchIt* package.[23] Additionally, we assessed the relationship of the disease-specific network and the healthy DMN in each group with individual patient cognitive dysfunction ratings. To this end, we calculated Spearman rank correlations for Mini-Mental State Examination (MMSE) scores with the respective network expression levels. In addition, we computed Delta, which we defined as the difference between the respective subject scores. We hypothesized that the MMSE correlations would be greater in magnitude for the difference measure than for the expression of either network alone. Correlations of network expression and Delta with MMSE were assessed by computing Spearman correlation coefficients. To determine whether the respective correlation coefficients computed in each disease group were significantly different, we applied the Steiger $z$-test (one-tailed). Correlations and Steiger tests were considered significant for $p < 0.05$. Only individuals with complete observations were included in these analyses. Correlation analysis was not performed in the MSA group because the MMSE data were not available in these subjects. All statistical analyses were conducted in RStudio version 2024.4.2.764, R version 4.4.1.

*Standard protocol approvals, registrations, and patient consents*

All contributing sites obtained local IRB/ethics approval for retrospective sharing and analysis of de-identified imaging data; written informed consent was obtained from all participants. ADNI procedures were approved by participating institutional IRBs, with written informed consent from all participants (details per ADNI policies).

*Data availability*

ADNI and AIMN data are available upon approval from the respective committees; other pooled data are available upon request from the authors under a data-sharing agreement.

### Results

*Category 1*

Category 1 disorders, which included bvFTD, PSP, and CBD were distinguished by substantial spatial overlap (DSC ≥ 0.30) of the respective disease networks (bvFTDRP, PSPRP, and CBDRP) and the healthy DMN topography (Figure 1A) and voxel-wise correlations (VWC) ($p < 0.001$, corrected for spatial autocorrelation) (Figure 1B, C). The closest topographic similarity to DMN was observed for bvFTDRP (DSC: 0.55; VWC: $R^2 = 0.45$ $p < 0.001$, corrected for spatial autocorrelation), with strong correlations between the respective expression values ($r = –0.87$, $p < 0.0001$; Pearson correlation) (Figure S1). PSPRP was also topographically similar to DMN (DSC: 0.44; VWC: $R^2 = 0.42$ $p < 0.001$), with tight correlations between the corresponding subject scores ($r = –0.92$, $p < 0.0001$) (Figure S1). Similar topographic relationships, although somewhat weaker, were also observed for CBDRP and the healthy DMN (DSC: 0.39; VWC: $R^2 = 0.33$ $p < 0.001$), and between corresponding expression values ($r = –0.82$, $p < 0.0001$) (Figure S1) in the CBD population. Indeed, the abnormal elevations in disease network expression observed in each of these disorders were accompanied by commensurate reductions in DMN subject scores ($p < 0.0001$; Student's *t*-tests) (Figure 2). In this vein, comparison of the expression trajectories of the pathological and healthy networks in Category 1 disorders revealed similar changes with advancing disease (Figure 3, *violet cluster*). Given the association of the above-mentioned Category 1 disorders with 4R tauopathy, we examined analogous network relationships in XDP, a rare neurodegenerative disorder in which tauopathic changes have recently been reported (see Introduction). Significant topographical overlap of the disease-specific XDPRP with the healthy DMN was also observed (DSC: 0.33; VWC: $R^2 = 0.15$, $p < 0.001$), as well as moderate correlation between the corresponding subject scores ($r = -0.49$, $p < 0.006$) (Figure S1).

*Category 2*

These disorders, exemplified by PD, AD, and DLB were associated with pathological networks that exhibited less overlap with the healthy DMN than those in Category 1. In PD, for example, the disease-specific PDRP topography had a smaller similarity coefficient and a non-significant voxel-wise correlation with DMN (DSC: 0.23; VWC: $R^2 = 0.06$, $p = 0.12$), with a moderate correlation between the respective subject scores ($r = -0.51$, $p < 0.001$). That said, the later-appearing PD-cognition related pattern (PDCP)[19] showed significant topographic relationships with DMN (DSC: 0.44; VWC: $R^2 = 0.48$, $p < 0.001$), and a stronger inverse correlation between the respective subject scores ($r = -0.66$, $p < 0.001$). Subtle loss of healthy DMN expression was observed in non-demented PD patients despite significant elevation in PDRP levels (Figure 2A, *middle panel*). Greater DMN reductions and more pronounced PDRP elevations were present in PDD patients compared to their non-demented PD counterparts. Even so, correlations between expression levels for the two topographies were similar in the demented and non-demented PD groups (Figure 3, *orange cluster*). An analogous relationship was observed between increasing DLBRP and declining healthy DMN subject scores in DLB. Nonetheless, the spatial overlap between the disease-specific and healthy topographies was marginal (DSC: 0.16; VWC: $R^2 =$

0.002, $p = 0.70$) as was the correlation of the respective expression values ($r = –0.18$, $p = 0.02$) (Figure S1). In DLB, DMN loss was minimal compared to the significant elevations in DLBRP expression that were observed (Figure 2).

Regarding the prodromal syndromes, iRBD patients showed significant PDRP elevations without abnormal reductions in DMN (Figure 2). Likewise, significant DLBRP elevations were observed in MCI-LB, with only minimal DMN reductions in the same individuals (Figure 2). Indeed, we found that estimated linear relationships between increasing PDRP and DLBRP expression and healthy DMN loss were similar for all Lewy body disorders, even at prodromal stages (Figure 3, *orange cluster*). Likewise, ADRP, a reproducible disease-specific topography independently identified in multiple AD populations, had comparatively low topographic similarity to DMN (DSC: 0.19; VWC: $R^2 = 0.03$, $p = 0.42$) (Figure 1B, C). Indeed, the slope of the line relating the respective subject scores in the AD sample was intermediate between the Lewy body disorders and the major primary tauopathies in Category 1 (Figure 3, *orange cluster*). In MCI-AD, we observed relatively greater increases in ADRP expression compared to healthy DMN loss (Figure 2). As with iRBD and MCI-LB, the linear estimate of the relationship between increased pathological network expression and DMN loss was similar in the prodromal and manifest disease groups (Figure 3, *orange cluster*).

*Category 3*

This category included MSA, for which the disease network was topographically unrelated to the healthy DMN. Indeed, no spatial overlap was observed between the pathological and healthy topographies (DSC: 0.03; VWC: $R^2 = 0.06$, $p = 0.25$) (Figure 1). Despite elevated MSARP expression in these patients, DMN values did not differ significantly from normal ($p = 0.50$)

(Figure 2). This accords with the trajectory of network expression estimated for this group (Figure 3, *blue cluster*).

*Clinical correlations*

In each group, we examined correlations between MMSE scores and standardized expression values for the relevant disease network and the healthy DMN, and for Delta, the difference between these values (see Methods). These correlations are provided in Supplementary Table 3. In bvFTD, MMSE correlated significantly with bvFTDRP ($\rho(76) = -0.36$, $p = 0.001$) and DMN ($\rho(76) = 0.40$, $p = 0.0003$) expression. Delta also correlated significantly with MMSE ($\rho(76) = -0.38$, $p < 0.01$), although this correlation was not different from that based on bvFTDRP expression alone (one-sided Steiger test: $t(75) = 0.99$, $p = 0.16$). In the other Category 1 disorders, neither disease network nor healthy DMN subject scores, and the difference in these values, correlated significantly with MMSE ($p > 0.09$).

In PD and PDD, MMSE did not significantly correlate with PDRP expression ($\rho(32) = -0.26$, $p = 0.14$), although a correlation with DMN scores was noted ($\rho(32) = 0.37$, $p = 0.03$). Delta correlated inversely with this cognitive measure ($\rho(32) = -0.38$, $p = 0.03$; Spearman correlations). Indeed, the MMSE correlation with Delta was significantly stronger than with PDRP scores alone ($t(31) = 2.18$, $p = 0.02$). In AD and MCI-AD, MMSE correlated with ADRP expression ($\rho(275) = -0.29$, $p < 0.01$), DMN scores ($\rho(275) = 0.275$, $p < 0.01$), and Delta ($\rho(275) = -0.36$, $p < 0.01$). Delta in this group exhibited a significantly stronger correlation with cognitive dysfunction than ADRP expression alone ($t(274) = 2.46$, $p = 0.008$). Likewise, in DLB and MCI-LB, DLBRP expression ($\rho(146) = -0.42$, $p < 0.01$), DMN ($\rho(146) = 0.31$, $p < 0.01$), and Delta ($\rho(146) = -0.47$, $p < 0.01$) correlated significantly with MMSE, and the association with Delta was significantly stronger ($t(145) = 1.68$, $p = 0.048$).

### Discussion

Based on our analysis of previously characterized disease networks and the healthy DMN, we assigned over 1,200 patients with neurodegenerative disorders in three distinct categories: those with imaging evidence of primary involvement of the healthy DMN (bvFTD, PSP, CBD, and XDP), those with secondary DMN loss (AD, PD, and DLB in manifesting and their prodromal stages), and those without evidence of DMN involvement (MSA).

*Category 1: Disorders with imaging evidence of primary DMN involvement*

bvFTD, PSP, and CBD are associated, although not exclusively by 4R tau pathology. Even though the clinical spectrum is broad, all three disorders are associated with some degree of dysexecutive cognitive decline.[24] The data suggest that bvFTD, PSP, and CBD belong to a distinct class of neurodegenerative disorders characterized by significant loss of the healthy DMN topography in the setting of prominent increases in pathological network expression. Thus, based upon the close topographic similarity of the disease network to the healthy DMN, these diseases can be viewed as a distinct group designated as Category 1. On the subject level, the increases in expression levels for the respective disease networks occur in parallel with reductions in healthy DMN activity (Figure 3, *violet cluster*). That said, characteristic disease networks typically include non-DMN regions in which brain dysfunction develops as part of the underlying pathological process. For example, bvFTDRP includes the caudate, thalami, temporal poles, and insula,[25] regions that do not contribute to the healthy DMN topography.[6] DMN connectivity changes, including anterior DMN disruption, are common but heterogeneous in bvFTD.[26,27] PSPRP, likewise, involves regions not included in the DMN, such as the thalamus and the striatum. Despite marked clinical heterogeneity, PSP subtypes share a common metabolic pattern.[28,29] Whether these differ in DMN involvement remains unknown.

Loss of functional connectivity in the DMN has been described recently in CBD and PSP.[30] We note that these disorders are pathologically distinct in that they represent the most common primary four-repeat (4R) tauopathies.[31] Interestingly, XDP, a rare neurodegenerative disorder in which accumulation of hyperphosphorylated 4R tau has recently been reported in brain tissue,[2] also showed significant although less extensive topographic overlap with the healthy DMN, as well as an inverse correlation between corresponding expression values. Despite the comparatively high similarity coefficient and significant voxel-wise correlation of the XDPRP topography with the healthy DMN (Figure 1B, C), the associated expression trajectory lay closer to those for Category 2 (Figure 3). Based on these observations, we assigned XDP to a transitional category (Category 1b). Given the close topographic and subject-level relationships that were observed between the various disease-networks and the healthy DMN, it is not surprising that DMN expression added little additional information to MMSE correlations in Category 1 patients.

*Category 2: Disorders with imaging evidence of secondary DMN involvement*

Metabolic imaging data from patients with Lewy body disorders (PD, PDD, and DLB) and AD, and their associated prodromal syndromes, point to DMN dysfunction occurring secondary to the underlying pathology. From a functional network standpoint, these disorders constitute a distinct disease category. At the time of diagnosis, PD is associated primarily with motor symptoms, whereas cognitive functioning is relatively preserved.[32] The earliest disease network to appear is

PDRP, for which expression levels track disease progression in the years before and after clinical onset.[18] Later, as the disease progresses, patients can develop MCI, which often leads to dementia (PDD).[32] That said, the linear trajectory that linked increasing PDRP expression to DMN loss was similar in the prodromal phase of the disorder (i.e., in iRBD) and in manifesting patients with or without cognitive dysfunction (Figure 3, *orange cluster*). Thus, in Category 2 disorders such as PD, the underlying disease process appears to be well underway by the time the pathology affects the healthy DMN.[33,34]

From a topographical standpoint, DLBRP is closely related to PDRP and to some extent also ADRP.[35] This accords with recent data showing that in DLB, cognitive impairment is explained by individual differences in subject scores for ADRP as well as DLBRP.[35] While DMN loss has been noted in a recent systematic review of DLB, this finding has been inconsistent.[36] In aggregate, the findings suggest that the DMN is unlikely to represent the primary network involved in DLB. In this vein, we noted analogous disparities between changes in disease network expression and healthy DMN activity in prodromal syndromes associated with Lewy body disorders. For example, in patients with iRBD, a prodromal condition for Lewy body disorders, PDRP elevations were present at baseline and increased steadily over time in the period before phenoconversion.[18,37] Indeed, an analogous relationship between DLBRP and DMN expression was observed in MCI-LB, a prodromal syndrome associated with future phenoconversion to DLB.[13,38]

AD and its clinical prodrome MCI-AD were also classified as Category 2 disorders. In AD, we observed significant elevations in ADRP expression ($p < 0.001$), while DMN scores were only modestly reduced compared to the corresponding average across Category 1 disorders (AD: 2.6 and –1.3; Category 1: 4.2 and –2.7, for the respective mean increase in the respective disease-related pattern expression and the associated reduction in DMN). These observations are consistent with two previous studies showing that DMN loss is predominantly age-related in the prodromal stage of the illness, becoming an important contributor to cognitive decline only after the development of overt dementia.[6,20] The findings suggest that the DMN reductions observed in AD represent secondary involvement of the healthy network that worsens with advancing disease. Studies with rs-fMRI in AD patients are in line with our observations, showing consistent DMN abnormalities at the dementia stage and varying results beforehand,[39,40] Along similar lines, cascading network failure in AD places the initial stressor in medial temporal lobe structures, with subsequent involvement of the posterior DMN.[41,42] The relationship of the medial temporal lobe to the DMN is not straightforward. Indeed, rs-fMRI studies suggest that hippocampal activity is not consistently coupled to the DMN,[9] and metabolic covariance analysis does not reveal a significant contribution of this region to the latter network topography.[6] That said, MMSE correlations in AD and MCI-AD were strengthened by computing Delta as opposed to the expression of ADRP alone. Indeed, the increase in pathological network expression relative to the loss of healthy DMN activity provides insights into cognitive progression in Category 2 disorders that are not available with each network individually.

*Category 3: Disorders without imaging evidence of DMN involvement*

MSA, a progressive α-synucleinopathy, is clinically characterized by autonomic failure, parkinsonism, and cerebellar involvement. MSA features oligodendroglial α-synuclein pathology distinct from PD and PDD, and cognitive impairment is unusual in this disorder.[43] Indeed, the MSARP topography is distinct from DMN, with no significant group-level evidence of DMN dysfunction.[44] Of note, MSA patients with cerebellar and parkinsonian phenotypes express topographically similar disease patterns.[45] It is not known, however, whether the same topography is expressed in the rare MSA patients with significant cognitive impairment. However, DMN scores in the lowest quartile of patients with MSA overlapped those at the low end of the Category 2 α-synucleinopathies (Figure 2A). Whether this subset of MSA patients has demonstrable cognitive dysfunction in addition to motor and/or autonomic dysfunction is uncertain. Even so, the data support the view of MSA as a distinct category of neurodegenerative proteinopathy in which significant DMN dysfunction is commonly not seen.

*Therapeutic implications*

The proposed classification scheme has implications beyond nosology. In AD, several treatments such as those targeting brain amyloid plaques are currently approved for clinical use. Likewise, novel disease-modifying therapeutics are currently being investigated for a variety of neurodegenerative disorders, mainly in Category 2. Such interventions are particularly relevant at early clinical/prodromal stages of these disorders when significant DMN involvement may still be limited. In this regard, disease modification can be demonstrated by objective slowing of disease network progression.[18,46] Ideally, successful disease-modifying therapies should also maintain the activity of the DMN and other healthy RSNs at baseline levels. In this regard, the dual network approach that we describe may provide useful information about disease status not available otherwise.

*Future directions and limitations*

Several limitations should be considered. While the retrospective, multicenter design provided us with a large total sample, it also introduces potential selection bias and heterogeneity in PET

acquisition and reconstruction. Although network expression scores were standardized to healthy controls, residual effects of sites may have influenced comparisons. Diagnostic uncertainty also varied across groups/sites, and clinically defined syndromes may encompass different underlying pathologies. Accordingly, the proposed categories should be interpreted as imaging patterns observed in the cohorts rather than definitive classifications of the respective diseases.

There is still ongoing discussion regarding the standardization of network-based nomenclature with rs-fMRI, in which the number of unique RSNs has been found to vary considerably.[47] As the field evolves, additional network homologies will likely be discovered to explain individual clinical phenotypes and to assess their corresponding progression rates. Notably, we chose the most common neurodegenerative proteinopathies as a starting point and the scheme should be validated and extended to additional disorders/phenotypes as more imaging data becomes available. Likewise, the data presented are derived mainly from cross-sectional studies, from which the temporal sequence of disease network alterations and DMN involvement can only be inferred. It is likely, however, that analogous changes will be detected in longitudinal analyses, with increases in disease network expression accompanied by reductions in healthy DMN activity.[6,18–20] Nonetheless, the utility of this dual network approach in disease modification trials remains to be determined.

*Conclusions*

Based on these findings, we propose a network-based nosology that stratifies major neurodegenerative disorders into three distinct categories according to the homology of previously validated disease-related topographies and the healthy DMN. Category 1 includes bvFTD, PSP, CBD, and XDP: disorders in which disease-specific metabolic topographies overlap considerably with the healthy DMN. These diseases are characterized by substantial overlap between each of the pathological networks and DMN, and inverse correlations between corresponding patient expression levels. Category 2 includes PD, DLB, and AD, and their associated prodromal syndromes. For these disorders, the characteristic disease topographies had limited spatial overlap with DMN, with smaller reductions in expression values for the latter compared to the corresponding pathological network. Indeed, in this category, disease-specific topographies were already established at prodromal stages of the illness. Although significant DMN reductions were not detected in iRBD or MCI-LB patients, such changes were observed in MCI-AD, which were smaller in magnitude than the corresponding ADRP elevations. This suggests that DMN involvement in this class of neurodegenerative disorders is likely to represent a secondary phenomenon. Interestingly, in Category 2, DMN scores provide additional information about cognitive impairment beyond disease network expression values. Category 3, exemplified by MSA, comprises disorders in which pathological networks emerge without consistent DMN involvement. In aggregate, the data support a dual-network imaging framework that can assist in the classification and monitoring of individual patients over time and in response to treatment.

**Acknowledgments**

The authors thank Drs. Martin Niethammer, Chris Tang, and Vijay Dhawan (Manhasset, NY, USA); Ji Hyun Ko (Winnipeg, Canada); Joachim Brumberg and Philipp T. Meyer (Freiburg, Germany); Timo Grimmer, Igor Yakushev, and Janine Diehl-Schmid (Munich, Germany); Madhavi Tripathi (New Delhi, India); Andrea Pilotto and Alessandro Padovani (Brescia, Italy); Klaus L. Leenders (Groningen, the Netherlands); Maja Trošt and Tomaž Rus (Ljubljana,

Slovenia); Chong Sik Lee (Seongnam, South Korea); and Valle Camacho (Barcelona, Spain) for sharing FDG PET data from their subjects. We thank the Italian Association of Nuclear Medicine (AIMN) and the following institutions and researchers for sharing the normal FDG dataset: Flavio Nobili and Silvia Morbelli, University of Genoa and IRCCS Ospedale Policlinico San Martino, Genoa; Sabina Pappatà, Institute of Bio-structure and Bioimaging of the CNR and Department of Advanced Biomedical Sciences, Federico II University, Naples; Angelina Cistaro, formerly at the IRMET, Torino, Italy; Duccio Volterrani, Regional Center of Nuclear Medicine, Hospital University of Pisa, Pisa; Valentina Berti, Nuclear Medicine Unit, University of Florence; Maria Lucia Calcagni, Nuclear Medicine Unit, Università Cattolica del Sacro Cuore, Rome; Stelvio Sestini, Nuclear Medicine Unit Santo Stefano Prato Hospital, Prato. We also thank Prof. Daniela Perani and Prof. Luca Presotto (Vita-Salute San Raffaele University, Nuclear Medicine Unit, San Raffaele Hospital, Division of Neuroscience, San Raffaele Scientific Institute, Milan) for the fruitful collaboration and for important work devoted to elaborating FDG data for the database. Finally, we thank Prof. Andrea Chincarini (Instituto Nazionale Fisica Nucleare, Genova) for the support in collecting and reviewing PET data. The authors are grateful to Ms. Yoon Young Choi for her valuable editorial assistance in preparing the manuscript, and to Drs. Sebastiano Vacca and Joshua J. Strohl for their thoughtful comments.

**Author Contributions**

**Matej Perovnik:** Conceptualization, Data curation (literature search), Formal analysis, Visualization (figures), Writing – original draft, Writing – review and editing. **David Eidelberg:** Conceptualization, Supervision (analysis oversight), Writing – review and editing.

**Study Funding**

This work was supported by The Slovenian Research Agency (ARRS) through grants P1-0389 and J3-60059 and by the National Institute of Neurological Disorders and Stroke R01 NS105979 and P50 NS071675 [Morris K. Udall Center of Excellence for Parkinson's Disease Research at The Feinstein Institute for Medical Research] to D.E. The XDP study was funded by the Collaborative Center for X-Linked Dystonia-Parkinsonism at the Massachusetts General Hospital. M.P. is a recipient of the Fulbright Visiting Scholar Program sponsored by the US Department of State's Bureau of Educational and Cultural Affairs. Data collection and sharing for this project was funded by the Alzheimer's Disease Neuroimaging Initiative (ADNI) (National Institutes of Health Grant U01 AG024904) and DOD ADNI (Department of Defense award number W81XWH-12-2-0012). ADNI is funded by the National Institute on Aging, the National Institute of Biomedical Imaging and Bioengineering, and through generous contributions from the following: AbbVie, Alzheimer's Association; Alzheimer's Drug Discovery Foundation; Araclon Biotech; BioClinica, Inc.; Biogen; Bristol-Myers Squibb Company; CereSpir, Inc.; Cogstate; Eisai Inc.; Elan Pharmaceuticals, Inc.; Eli Lilly and Company; EuroImmun; F. Hoffmann-La Roche Ltd and its affiliated company Genentech, Inc.; Fujirebio; GE Healthcare; IXICO Ltd.; Janssen Alzheimer Immunotherapy Research & Development, LLC.; Johnson & Johnson Pharmaceutical Research & Development LLC.; Lumosity; Lundbeck; Merck & Co., Inc.; Meso Scale Diagnostics, LLC.; NeuroRx Research; Neurotrack Technologies; Novartis Pharmaceuticals Corporation; Pfizer Inc.; Piramal Imaging; Servier; Takeda Pharmaceutical Company; and Transition Therapeutics. The Canadian Institutes of Health Research is providing funds to support ADNI clinical sites in Canada. Private sector contributions are facilitated by the Foundation for the National Institutes of Health

(www.fnih.org). The grantee organization is the Northern California Institute for Research and Education, and the study is coordinated by the Alzheimer's Therapeutic Research Institute at the University of Southern California. ADNI data are disseminated by the Laboratory for Neuro Imaging at the University of Southern California.

**Disclosure**

The authors have declared that no conflict of interest exists.

### Figure Legends

**Figure 1. Topographical relationships of disease-related metabolic networks and the normal default mode network (DMN). (A)** Visualization of DMN (*left*) and individual disease networks displayed by category (see text). Hypometabolic regions are color-coded blue to green, and relatively hypermetabolic regions are color-coded red to yellow. **(B)** Dice-Sørensen similarity coefficients[21] for DMN with the respective disease-related networks. **(C)** Voxel-wise correlation plots of the respective region weights; p-values were corrected for spatial autocorrelation[22] (see Methods). [DMN – default mode network, bvFTDRP – frontotemporal dementia-related pattern, PSPRP – progressive supranuclear palsy-related pattern, CBDRP – corticobasal degeneration-related pattern, XDPRP – X-linked dystonia-parkinsonism-related pattern, PDRP – Parkinson's disease-related pattern, ADRP – Alzheimer's disease-related pattern, DLBRP – dementia with Lewy bodies-related pattern, MSARP – multiple system atrophy-related pattern.]

**Figure 2. Expression levels for the default mode network (DMN) and for the disease-related metabolic networks in patients and matched healthy subjects. (A)** Disease-specific pattern expression was significantly elevated for all patient groups compared to samples of healthy control (HC) subjects matched for age and sex to the corresponding disease group (see Methods). **(B)** DMN expression was significantly lower for all groups ($p < 0.0001$) except for iRBD ($p = 0.13$), MCI-LB ($p = 0.24$), and MSA ($p = 0.50$) compared to HC subjects (see text). [***$p < 0.0001$, *$p < 0.05$, ns – non-significant. bvFTD – behavioral variant frontotemporal dementia, PSP – progressive supranuclear palsy, CBD – corticobasal degeneration, XDP – X-linked dystonia-parkinsonism, PDD – Parkinson's disease dementia, PD – Parkinson's disease, iRBD – idiopathic rapid eye movement sleep behavior disorder, AD – Alzheimer's dementia, MCI-AD –

mild cognitive impairment due to Alzheimer’s disease, DLB – dementia with Lewy bodies, MCI-LB – mild cognitive impairment with Lewy bodies, HC – healthy control, MSA – multiple system atrophy, DMN – default mode network, bvFTDRP – frontotemporal dementia-related pattern, PSPRP – progressive supranuclear palsy-related pattern, CBDRP – corticobasal degeneration-related pattern, XDPRP – X-linked dystonia-parkinsonism-related pattern, PDRP – Parkinson’s disease-related pattern, ADRP – Alzheimer’s disease-related pattern, DLBRP – dementia with Lewy bodies-related pattern, MSARP – multiple system atrophy-related pattern.]

**Figure 3. Relationships between expression levels for disease-related networks and healthy default mode network (DMN) in the patient groups.** For each group, mean expression values for the normal DMN and the associated disease-specific pattern are represented by an arrowhead. The dashed line indicates equal-magnitude disease-related pattern elevation and DMN reduction. Points above this line indicate greater disease-related pattern elevation than DMN reduction; conversely points below the line would indicate greater DMN loss than gain in disease network expression. [bvFTD – behavioral variant frontotemporal dementia, PSP – progressive supranuclear palsy, CBD – corticobasal degeneration, XDP – X-linked dystonia-parkinsonism, PDD – Parkinson’s disease dementia, PD – Parkinson’s disease, iRBD – idiopathic rapid eye movement sleep behavior disorder, AD – Alzheimer’s dementia, MCI-AD – mild cognitive impairment due to Alzheimer’s disease, DLB – dementia with Lewy bodies, MCI-LB – mild cognitive impairment with Lewy bodies, MSA – multiple system atrophy, DMN – default mode network.]

**Figure 1.** Topographical relationships of disease-specific related metabolic networks and the normal default mode network (DMN).

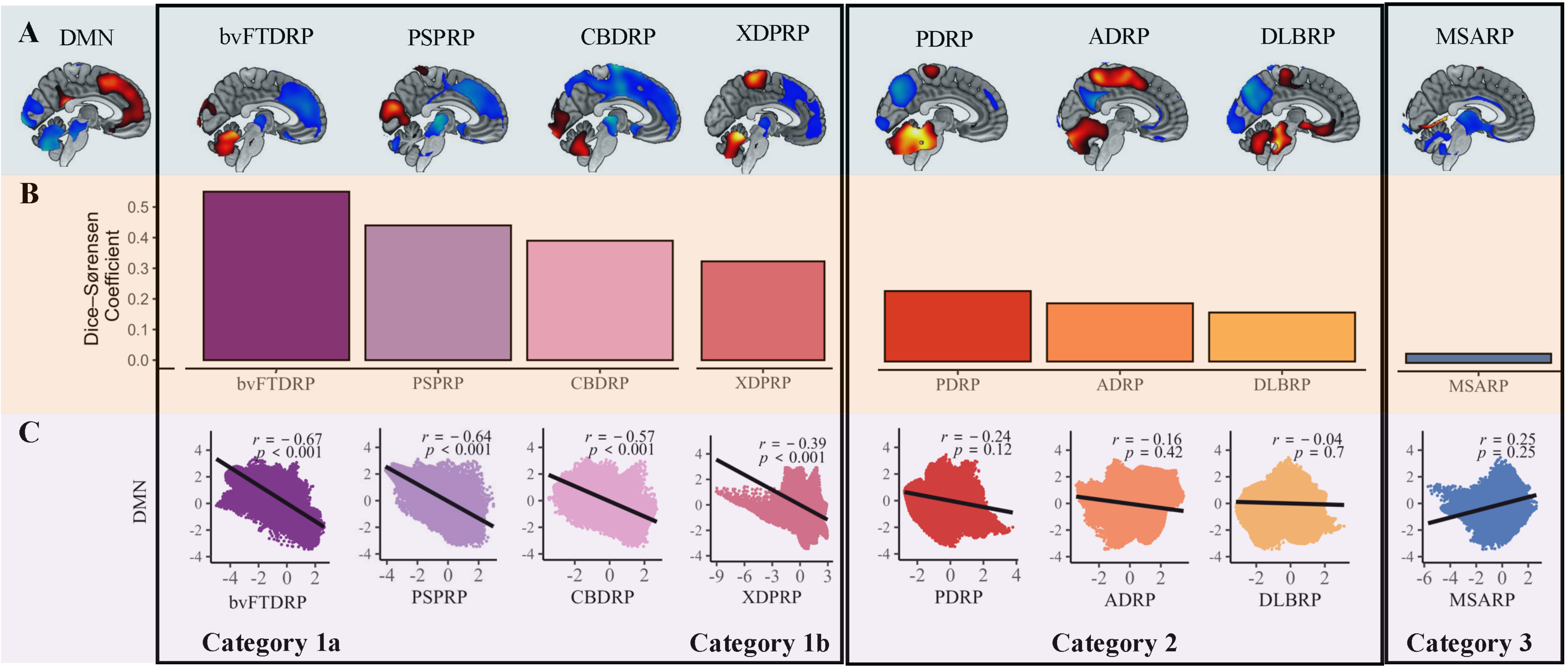

**Figure 2.** Expression levels for the default mode network (DMN) and for the disease-related metabolic networks in patients and matched healthy subjects.

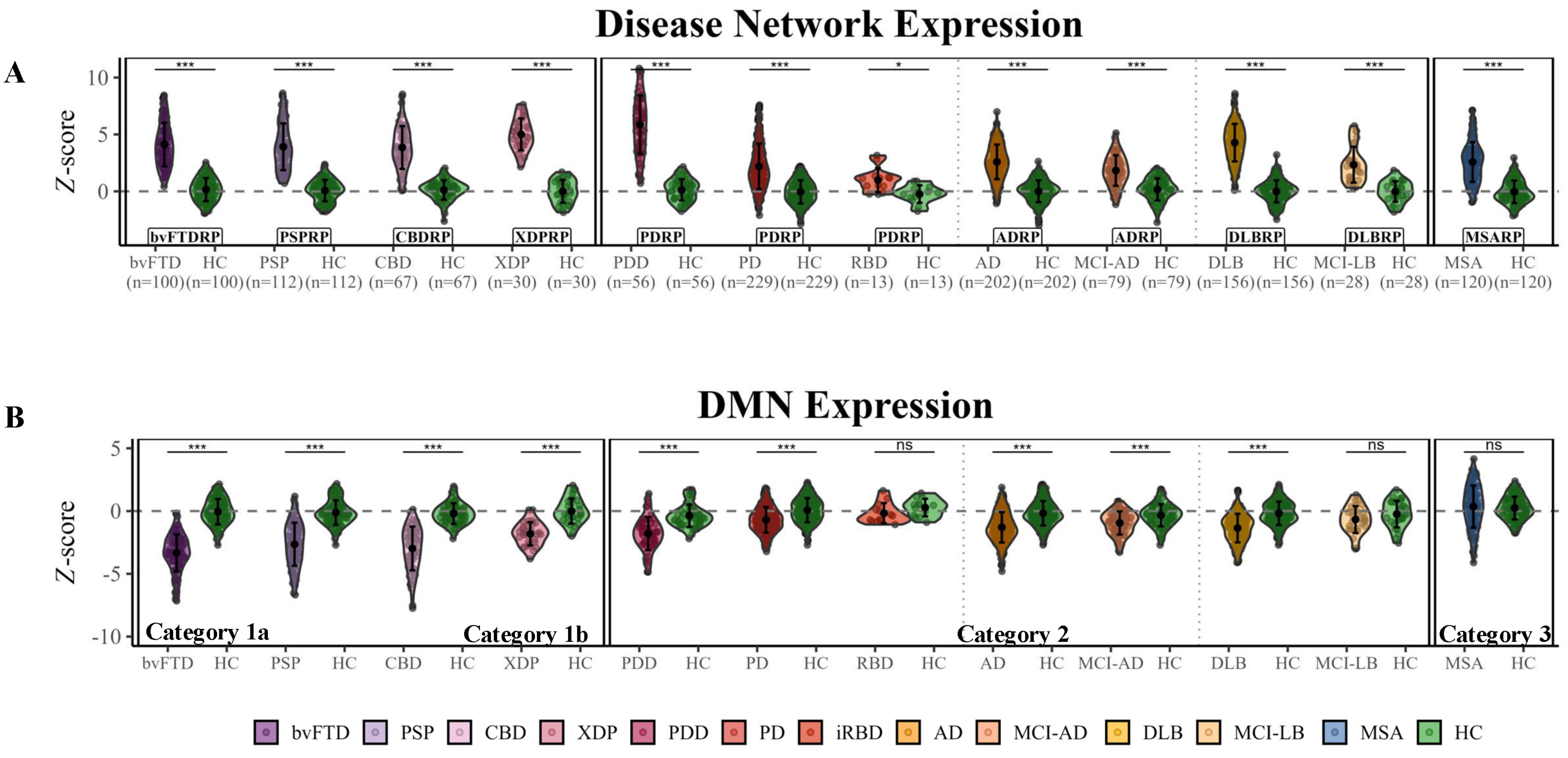

**Figure 3.** Relationships between expression levels for disease-related networks and healthy default mode network (DMN) in the patient groups.

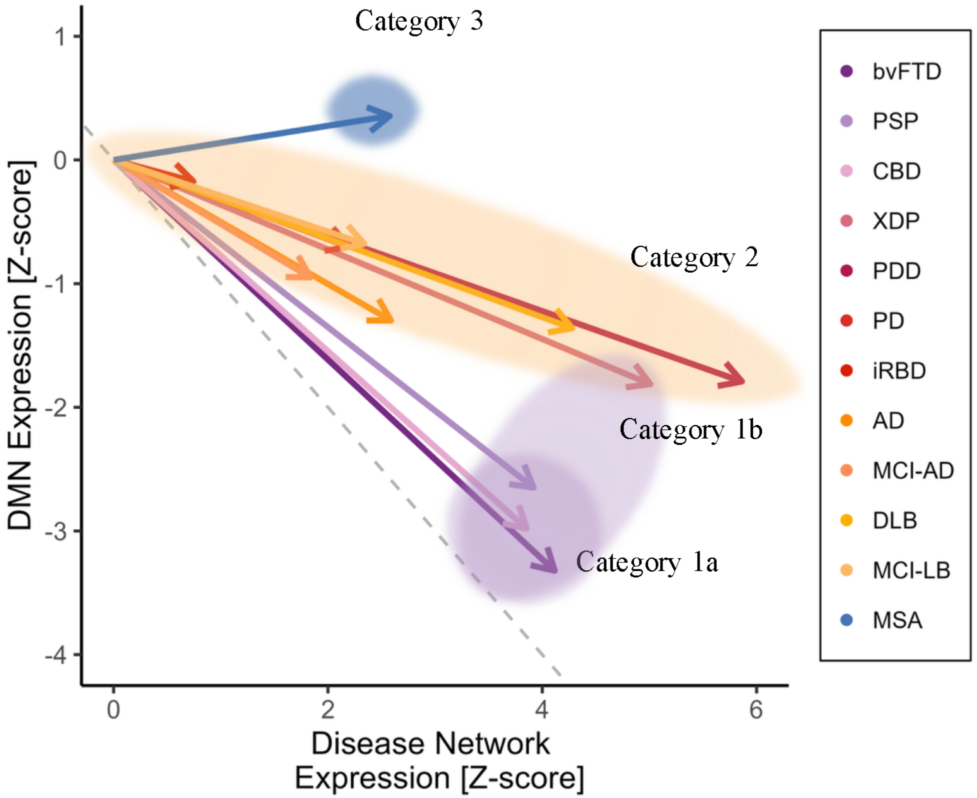

## Supplementary Files

This is a list of supplementary files associated with this preprint. Click to download.

- PerovnikEidelbergSupplMat.docx
